# Splenic peliosis with spontaneous splenic rupture: report of two cases

**DOI:** 10.1186/1471-2482-6-9

**Published:** 2006-06-26

**Authors:** Daniel J Lashbrook, Roger W James, Andrea J Phillips, Anthony G Holbrook, Andrew C Agombar

**Affiliations:** 1 Department of General Surgery, Royal United Hospital, Combe Park, Bath, UK; 2 Department of Cellular Pathology, Royal United Hospital, Combe Park, Bath, UK; 3 Department of Radiology, Royal United Hospital, Combe Park, Bath, UK

## Abstract

**Background:**

Peliosis is a rare condition characterised by multiple cyst-like, blood-filled cavities within the parenchyma of solid organs. Most commonly affecting the liver, isolated splenic peliosis is an even more unique phenomenon. Patients with the condition are often asymptomatic. However, this potentially lethal condition can present with spontaneous organ rupture. We present two such cases, discuss their management and review what is currently known in the existing literature.

**Case presentation:**

A previously well twenty-six year old woman presented with abdominal pain following a trivial episode of coughing. A diagnosis of spontaneous splenic rupture was made following clinical and radiological examination. She underwent emergency splenectomy and made a full, uneventful recovery. Histopathological examination confirmed splenic peliosis.

The second case describes an eighty six year old lady who sustained a trivial fall and developed pain in her left side. A CT confirmed splenic rupture. She became haemodynamically unstable during her admission and underwent emergency splenectomy. Histopathological examination revealed splenic peliosis. She went on to make an uneventful recovery.

**Conclusion:**

Splenic peliosis is very rare. It has a number of associations including immunosuppression, drug therapy and infection. Although patients are often asymptomatic, life-threatening spontaneous organ rupture may occur. If the diagnosis of peliosis is confirmed, additional investigations should be considered to detect its presence in other organs. Furthermore, the presence of the condition may be relevant if further medical or surgical intervention is planned.

## Background

Peliosis was first described in the liver (peliosis hepatis) by Schoenland in 1916 [1]. Most cases of splenic peliosis are associated with peliosis hepatis. Isolated splenic peliosis (peliosis lienis) is extremely rare. Its presence has only been reported thirty four times in the literature with only seven documented cases of organ rupture.

Establishing its incidence has proved difficult since the condition either remains asymptomatic or is discovered incidentally at autopsy [14]. Tada et al. examined the spleens of 1,200 random autopsy cases [2] over a three year period. The presence of peliosis was noted in ten patients and in eight of these cases it was confined to the spleen alone.

We present two patients with spontaneous splenic rupture and review the literature with regard to the surgical, pathological and radiological findings in such cases as well as the management of patients with this condition.

## Case presentation

### Case one

A previously healthy twenty-six year old woman was admitted with a five day history of influenza-like symptoms. She had consulted her general practitioner on the morning of admission after developing left loin pain with associated dysuria, nausea and vomiting. This pain appeared to follow a bout of coughing. A provisional diagnosis of pyelonephritis was made and she was prescribed an oral antibiotic. Her pain worsened and she was admitted to hospital that same afternoon.

She was noted to be taking the combined oral contraceptive pill. Her medical history was otherwise unremarkable.

Initial clinical examination revealed her to be comfortable lying flat. Her loin pain was exacerbated by movement. Her mucosal membranes were dry. Despite a blood pressure of 99/58 there were no other features of hypovolaemic shock. She was apyrexial.

Cardio-respiratory examination was unremarkable. She had tenderness in the left side of her abdomen with no associated features of peritonism. The remainder of her clinical examination was normal.

Her routine bloods tests were unremarkable. Epstein-Barr Virus Nuclear antigen antibody serology was positive suggesting previous exposure to the virus. A plain KUB radiograph and dip-stick urinalysis were negative. A pregnancy test was also negative.

Overnight her condition deteriorated. Her abdominal pain increased and she became hypotensive. Repeat clinical examination revealed a pulse of 78 beats per minute with a blood pressure of 82/40. She was now peritonitic in the left side of her abdomen. Rectal examination revealed a boggy mass in the pouch of Douglas.

**Figure 1:**
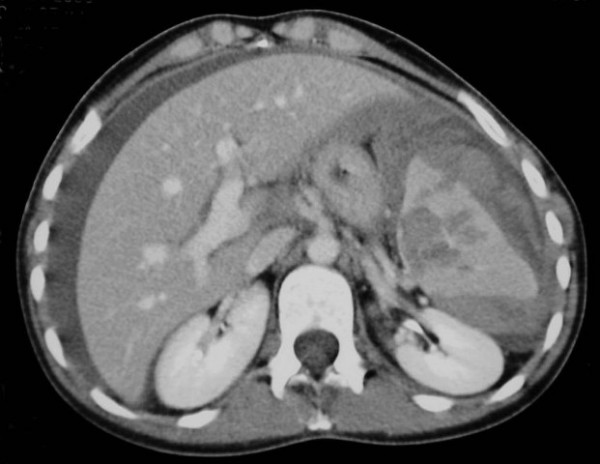
Case 1. Contrast-enhanced CT (CECT) demonstrates a splenic laceration extending to the splenic surface from a focal hypodense cyst-like lesion. There is a large perisplenic haematoma and abdominal free fluid.

**Figure 2:**
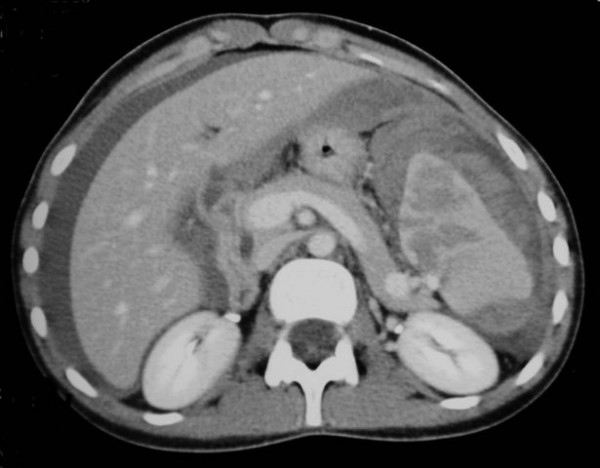
Case 1. Splenic vessels course through the cyst-like lesions of the splenic parenchyma without mass effect. The lesions show areas of contrast enhancement on the portal venous phase of CECT.

**Figure 3:**
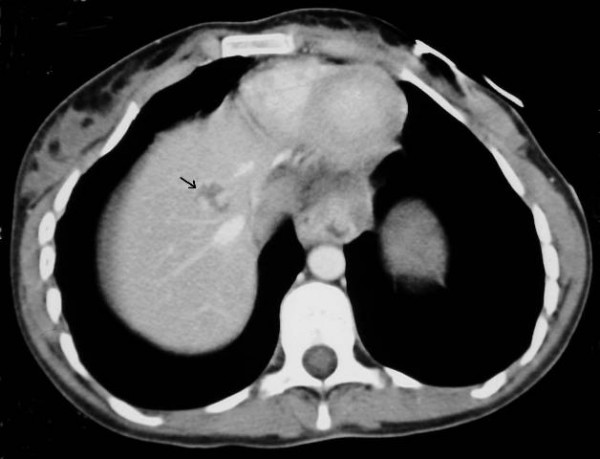
Case 1. CECT identified a 1.5 cm lesion of low attenuation in the dome of the right lobe of the liver (arrow). The lesion demonstrates nodular peripheral enhancement on the portal venous phase.

An urgent abdominal CT scan with intravenous contrast was arranged. This demonstrated extensive free fluid throughout the abdomen and a large perisplenic haematoma. The spleen had several lacerations extending to the surface from focal hypodense cyst-like lesions of the splenic parenchyma (Fig 1). These lesions contained foci of contrast enhancement and did not exert mass effect on the splenic vessels coursing through them (Fig 2). In addition, a 1.5 cm lesion of low attenuation, demonstrating nodular peripheral enhancement on the portal venous phase, was noted in the dome of the right lobe of the liver (Fig 3). The right ovary also contained a 3.5 cm area of low attenuation consistent with an ovarian cyst.

The patient was taken to the operating theatre where she underwent emergency splenectomy via a midline laparotomy. From within her peritoneal cavity, 1500 mls of free blood were evacuated. The spleen was found to be deeply lacerated with active bleeding. Post-operatively she was admitted to the Intensive Therapy Unit and returned to the ward after twenty-four hours. She went on to make a rapid and uneventful recovery and was discharged home on day six.

Gross examination showed a 250 g spleen with multiple subcapsular haemorrhagic nodules, one of which was associated with an area of capsular rupture. Sectioning revealed dilated vascular spaces measuring up to 10 mm in diameter present throughout the splenic parenchyma. Histological examination showed unlined, blood-filled spaces (Figs 6 and 7). There was red pulp congestion with scattered Warthin-Finkeldey polykaryocytes (Fig 8). In lymph nodes, these are associated with viral infection, in particular (but not specifically) measles. These features were consistent with splenic peliosis.

Her oral contraceptive medication was discontinued. A follow-up liver MRI scan was planned for further evaluation of the liver lesion to differentiate between a haemangioma and a solitary focus of hepatic peliosis. The MRI confirmed the lesion to be a haemangioma.

### Case two

An eighty-six year old female presented to the Accident and Emergency department. She tripped and fell at home three days prior to admission, landing on her left side. On admission she complained of left sided abdominal and chest pain. She was known to have atrial fibrillation, congestive cardiac failure and rheumatoid arthritis. In the past, she had received a short course of corticosteroids for her rheumatoid disease. She denied any history of immunosuppressive therapy.

Examination revealed a localised area of peritonism in her left upper quadrant. She was haemodynamically stable with a blood pressure of 124/74 and a pulse of 76. Her haematological profile revealed a total white cell count of 11.9 (4 – 11 × 10^9^/l), haemoglobin of 10.1 (11.5 – 15.5 g/dl) with a normal mean corpuscular volume. Biochemical analysis revealed a creatinine of 111 μmol/l (54 – 84) but was otherwise unremarkable. Her INR was 1.2. Liver function was within normal limits.

**Figure 4:**
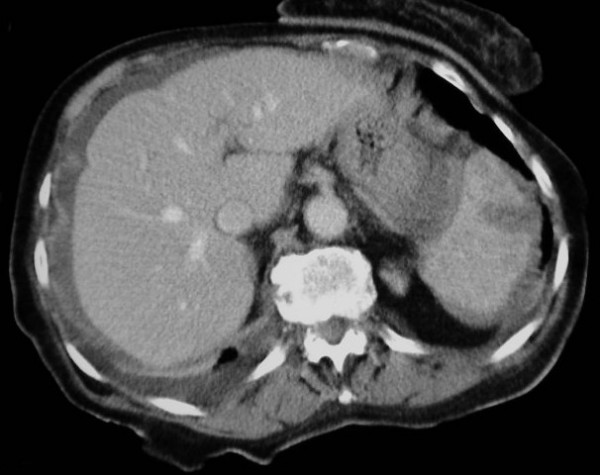
Case 2. CECT shows a laceration through the anterosuperior portion of the spleen with perisplenic haematoma and abdominal free fluid.

**Figure 5:**
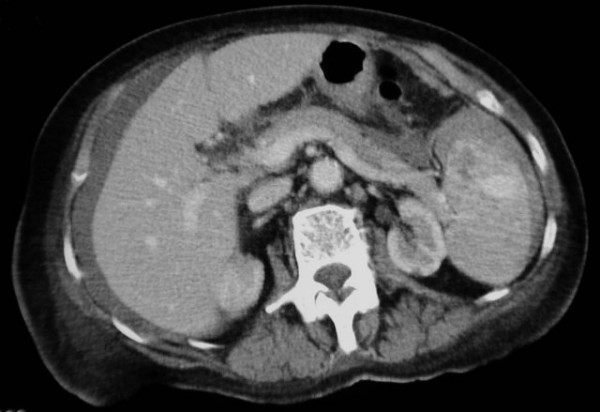
Case 2. Areas of contrast enhancement are seen more inferiorly in the spleen on CECT. These lie adjacent to a hypodense cyst-like lesion and further lacerations. The splenic capsule is disrupted anteriorly.

**Figure 6:**
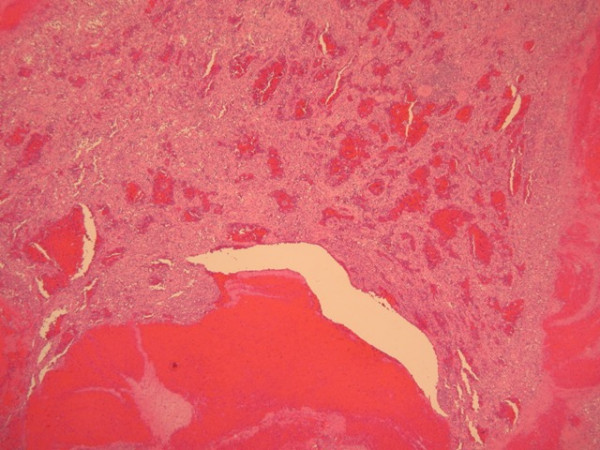
Case 1. Low power section demonstrating unlined, blood-filled spaces.

**Figure 7:**
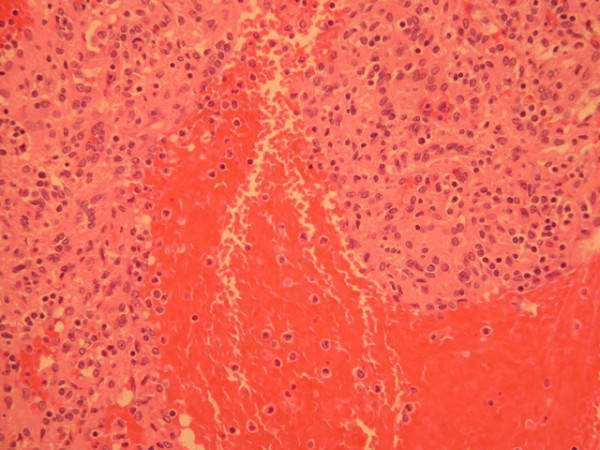
Case 1. High power section demonstrating unlined, blood-filled spaces.

**Figure 8:**
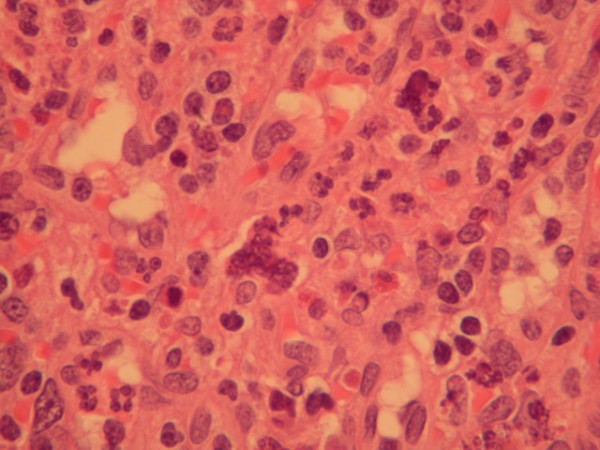
Case 1. Warthin-Finkeldey cells.

An urgent abdominal CT scan was requested. This demonstrated a complex laceration through the anterosuperior portion of the spleen extending to its lateral margin with perisplenic haematoma free intraperitoneal blood (Fig 4). More inferiorly the splenic parenchyma demonstrated foci of contrast enhancement adjacent to a hypodense cyst-like lesion and further lacerations, with disruption of the splenic capsule anteriorly (Fig 5)

She was initially managed conservatively on the ward but became haemodynamically unstable. In addition her haemoglobin was found to have dropped from 10.1 to 6.8. She subsequently underwent emergency splenectomy via midline laparotomy without complication. She went on to make a full recovery.

**Figure 9:**
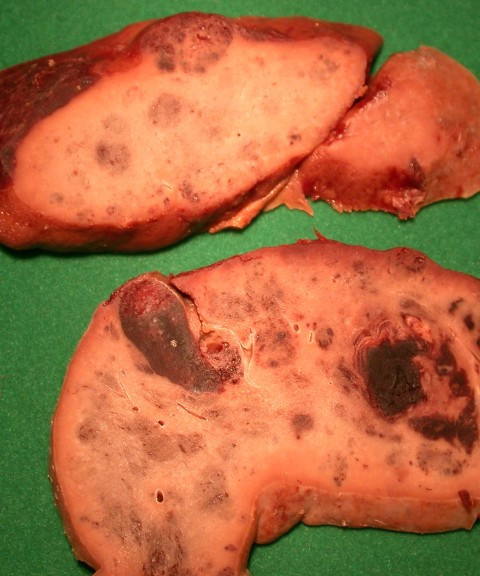
Case 2. Sectioned spleen showing multiple blood-filled spaces throughout the parenchyma.

**Figure 10:**
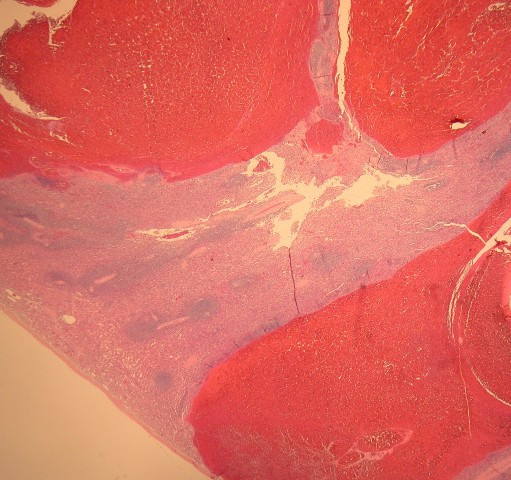
Case 2. Low power section demonstrating unlined, blood-filled spaces.

**Figure 11:**
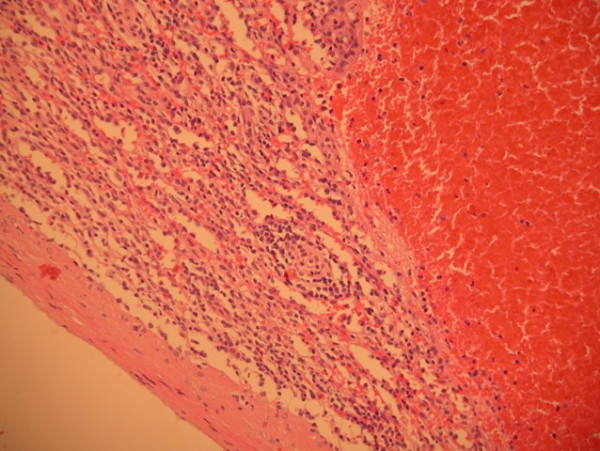
Case 2. High power section demonstrating unlined, blood-filled spaces.

Macroscopic examination showed a spleen measuring 110 × 85 × 40 mm weighing 215 g. Subcapsular haemorrhagic nodules were noted, one of which was associated with an 80 mm capsular tear (Fig 9). Sectioning revealed multiple blood-filled spaces up to 15 mm in diameter throughout the parenchyma. Histology showed unlined cystic spaces filled with blood (Figs 10 and 11). Many of these showed a parafollicular distribution. The features were consistent with splenic peliosis.

## Conclusion

Peliosis is a pathological entity characterized by the gross appearance of multiple cyst-like, blood-filled cavities within solid organs. It is traditionally thought that peliosis exclusively develops in organs belonging to the mononuclear phagocytic system (liver, spleen, bone marrow, and lymph nodes) [3]. However, a paucity of studies indicate that other organs such as the lungs, parathyroid glands, and kidneys may be affected too [4-6]. Isolated splenic peliosis was unreported until 1978 [11]. Until then it was thought to occur only in association with the more common peliosis hepatis[7].

Many aetiological agents have been postulated. Drugs and toxins including chronic alcoholism, corticosteroids, oral contraceptives, tamoxifen, azathioprine and androgens have been linked with the occurrence of peliosis [2,3,9,14]. The latter may help to explain why the disease appears to be more common in males [2]. The now-discontinued x-ray contrast medium Thorotrast, and vinyl chloride have also been implicated [1-9].

Patients with AIDS may develop peliosis in association with bacillary angiomatosis and parenchymal bacillary peliosis. This is due to secondary infection with *Bartonella henselae *and a similar organism, *Rochalimaea henselae *[1,8]. Both organisms can cause fever and abdominal pain [9]. Other infective agents such as hepatitis B and C [3,10,13], *Staphylococcus aureus *and tuberculosis are thought to be associated [1,2,9,12-14].

The development of peliosis is associated with both monoclonal gammopathies e.g. multiple myeloma and Waldenstrom's macroglobulinemia and other malignancies such as Hodgkin's disease, hepatoma and seminoma [9,12].

High circulating levels of vascular endothelial growth factor have also been linked. Joseph et al. describes on-going massive haemorrhagic ascites in a patient despite elective splenectomy [11]. Additionally, the condition has been described in patients with diabetes mellitus, end-stage renal disease, and renal transplantation [9].

The diversity of postulated aetiological agents shows how little is understood of this condition. A common theme in the existing literature is the link between peliosis and alterations in immune function. Most postulated causes are therefore centred around this.

The identification of peliosis can usually be made macroscopically, either at the time of surgery or during radiological investigations. It may also be detected at autopsy. The surface of the spleen may show nodular areas belying the underlying condition [7], but it is usually the cut surface that demonstrates the numerous cyst-like blood filled cavities. These lesions are usually seen as round or oval dilated sinuses in the parafollicular area of the red pulp. Simple dilatation of splenic sinuses caused by passive congestion is usually seen, by contrast, in the interfollicular area [2].

Occasionally arteries with their associated periarteriolar sheaths project into the lumen of the cysts. This may well contribute to the catastrophic and potentially life-threatening haemorrhage that can occur from affected organs.

Once the presence of peliosis has been established it is proposed that all necessary investigations be pursued to detect its presence in other organs and to establish a possible cause. The implications for this are clear. Spontaneous rupture of an affected organ remains an ever-present threat. Physicians should be mindful of this in the event that anticoagulation or thrombolysis is being considered. In addition, the presence of peliosis may prompt the investigation and subsequent discovery of equally serious underlying conditions, for example immunodeficiency or malignancy.

The finding of cyst-like hypodense lesions which do not cause mass effect on vessels on contrast-enhanced CT should alert the radiologist to the diagnosis of peliosis. This is of importance in recognizing the risk of spontaneous splenic rupture and in avoiding interventional procedures, such as liver biopsy or percutaneous transhepatic cholangiography, which may precipitate life-threatening iatrogenic organ rupture.

Haemorrhage associated with splenic peliosis is quite amenable to surgical therapy if recognised and treated promptly [15]. The existing literature does not however explore the surgical management of patients in whom an incidental diagnosis of splenic peliosis has been made. The introduction of laparoscopic splenectomy has reduced significant morbidity when compared with open surgery [16]. Prophylactic laparoscopic splenectomy has been advocated to prevent organ rupture in conditions such as splenic sarcoidosis [17]. The role of elective splenectomy in splenic peliosis is not known.

From the patient's perspective, lifestyle changes may be necessary. Younger patients should be encouraged to avoid high-risk activities, for example, contact sports. It would seem wise to avoid or discontinue the oral contraceptive pill, and seek alternative forms of contraception. In addition any attending physician should be aware when other medications thought to cause peliosis are being considered.

## Abbreviations

**CT **– computerised tomography

**KUB **– Kidney, Ureters, Bladder radiograph.

**MRI **– Magnetic Resonance Imaging

**HIV **– Human Immuno-deficiency Virus

**INR **– International Normalised Ratio

**AIDS **– Acquired Immune Deficiency Syndrome

## Competing interests

The author(s) declare that they have no competing interests.

## Authors' contributions

**DJL **assisted with the surgery of the first case, researched the literature, and drafted the manuscript.

**RWJ **made the histological diagnoses and prepared the pathology images. He also advised on the format and design.

**AJP **prepared the radiological images and advised on the format and design.

**ACA **performed the surgery for the first case.

All authors have reviewed and approved the final manuscript.

## Pre-publication history

The pre-publication history for this paper can be accessed here:
